# A Systemic Review of Functional Near-Infrared Spectroscopy for Stroke: Current Application and Future Directions

**DOI:** 10.3389/fneur.2019.00058

**Published:** 2019-02-05

**Authors:** Muyue Yang, Zhen Yang, Tifei Yuan, Wuwei Feng, Pu Wang

**Affiliations:** ^1^Department of Rehabilitation Medicine, Ruijin Hospital, Shanghai, China; ^2^School of Medicine, Shanghai Jiao Tong University, Shanghai, China; ^3^Core Facility of West China Hospital, Sichuan University, Chengdu, China; ^4^Shanghai Mental Health Centre, Shanghai Jiao Tong University School of Medicine, Shanghai, China; ^5^Department of Neurology, Medical University of South Carolina, Charleston, SC, United States

**Keywords:** near-infrared spectroscopy, stroke, motor recovery, cortical function, systemic review

## Abstract

**Background:** Survivors of stroke often experience significant disability and impaired quality of life. The recovery of motor or cognitive function requires long periods. Neuroimaging could measure changes in the brain and monitor recovery process in order to offer timely treatment and assess the effects of therapy. A non-invasive neuroimaging technique near-infrared spectroscopy (NIRS) with its ambulatory, portable, low-cost nature without fixation of subjects has attracted extensive attention.

**Methods:** We conducted a comprehensive literature review in order to review the use of NIRS in stroke or post-stroke patients in July 2018. NCBI Pubmed database, EMBASE database, Cochrane Library and ScienceDirect database were searched.

**Results:** Overall, we reviewed 66 papers. NIRS has a wide range of application, including in monitoring upper limb, lower limb recovery, motor learning, cortical function recovery, cerebral hemodynamic changes, cerebral oxygenation, as well as in therapeutic method, clinical researches, and evaluation of the risk for stroke.

**Conclusions:** This study provides a preliminary evidence of the application of NIRS in stroke patients as a monitoring, therapeutic, and research tool. Further studies could give more emphasize on the combination of NIRS with other techniques and its utility in the prevention of stroke.

## Introduction

Stroke, which refers to a medical condition in which insufficient brain blood supply results in cell death, is a major cause of death and disability worldwide (1, 2). Survivors are accompanied with the deterioration or loss of functions, for example, sensorimotor sequelae including motor weakness and impairment of voluntary motor control, spasticity, incoordination, apraxia, sensory loss/numbness, dysphagia, and dysarthria, and stroke could also lead to various cognitive and psychiatric deficits (3–5). These disfunctions are associated with cortical impairment due to insufficient blood supply and brain oxygenation. Therefore, monitoring the changes of brain circulation and oxygenation could timely reflect rehabilitation and recovery and the effect of therapy.

Neuroimaging has been shown to be an effective monitoring and therapeutic tool, evaluating the evolution of neural activity and stroke rehabilitation and recovery (6). Traditional methods such as functional magnetic resonance imaging (fMRI), positron emission tomography, electroencephalography (EEG) and magnetoencephalography (MEG)—have provided considerable initial insight into brain changes during recovery. However, several shortcomings including a confining monitoring environment, subject head fixation and high cost have limited applications in tasks which require constant movement or real-time monitor (7, 8).

Near-infrared spectroscopy (NIRS), introduced in 1977 by Jöbsis et al. as a monitoring tool of cerebral and myocardial oxygenation (9), has partially overcome these difficulties. NIRS is a non-invasive neuroimaging tool that has several potential advantages including real-time monitor, low price, simplicity, portability, relatively small equipment, and it's almost completely safe and non-invasive nature (8).

NIRS can be divided into continuous wave NIRS (CW NIRS), time domain NIRS (TD NIRS), and frequency domain NIRS (FD NIRS). CW NIRS emits continuous wave and measures the changes in the intensity of the light that passed through the tissue, whereas TD NIRS utilizes a short pulse of laser light and measures the arrival times of photons emerging from the tissue. FD NIRS records the intensity of the detected light as well as the phase shift. These signals can then be converted to the concentration of oxygenated (oxy-Hb) and deoxygenated hemoglobin (deoxy-Hb). One of the most common used algorithm is Modified Beer–Lambert Law (MBLL). CW NIRS could not measure absolute concentrations of oxy-Hb and deoxy-Hb, because this method assumes a homogenous tissue which is not true. This does not change the results of qualitative analysis, but may lead to error in quantitative outcome. TD NIRS recording the temporal broadening of the pulse as it penetrates the investigated area allows accurate quantification of the concentrations and has better spatial resolution (10). Neural activity increases oxygen demands, thus increasing cerebral blood flow due to neurovascular coupling. NIRS could capture the changes of oxy-Hb and deoxy-Hb to infer changes in brain activity (6, 11). Recent years have witnessed rapid development of the techniques of NIRS from the single-location measurements to two dimensions and then three dimensions. One of its most promising application is in brain-computer interface (BCI) which was firstly introduced by Coyle et al. (12). BCI use brain activity to control external devices bypassing the peripheral nervous system. NIRS presents as a valuable tool in its brain signal acquisition for its non-invasive nature and real-time monitoring. However, fNIRS-BCI system is still mainly used in researches due to slow information transfer rate and low accuracy (13).

Stroke includes two major types: ischemic, due to lack of cerebral blood flow, and hemorrhagic, due to bleeding. During ischemic stroke cerebral blood supply is disrupted by narrowing of vessels caused by atherosclerosis or embolism. In hemorrhagic stroke caused by hypertension or ruptured aneurysm blood flow is reduced due to direct blood loss or vessel compression. In either case, a significant decline in blood supply could be observed. The reduction in cerebral oxygenation results in injury of neurovascular unit, decreased neuronal activity and accumulation of anaerobic metabolites. NIRS could monitor oxygenation signal changes, thus reflecting this pathophysiological process (1).

NIRS is well-established as a safe and effective monitoring tool for stroke recovery, including upper limb, lower limb recovery, motor learning, cortical function recovery (8), cerebral hemodynamic changes, cerebral oxygenation (14), therapy (15, 16), clinical researches and evaluation of the risk for stroke (17). Brain-computer interfaces (BCIs), a newly emerging tool, use brain activity to control external devices, facilitating paralyzed patients to interact with the environment. NIRS combined with BCI offers great potential as a therapeutic tool (18, 19). In addition, NIRS has used in several clinical studies (20, 21), reflecting hemodynamic or oxygenation changes of brain, as well as in evaluation of the risks for postoperative stroke and muscle metabolism (22). NIRS has shown to be an effective and promising method, however, its clinical value still remains controversial (23). Therefore, to address these discrepancies, we conducted this systematic review to summarize the existing application of NIRS in stroke patients.

## Methods

**Figure 1:**
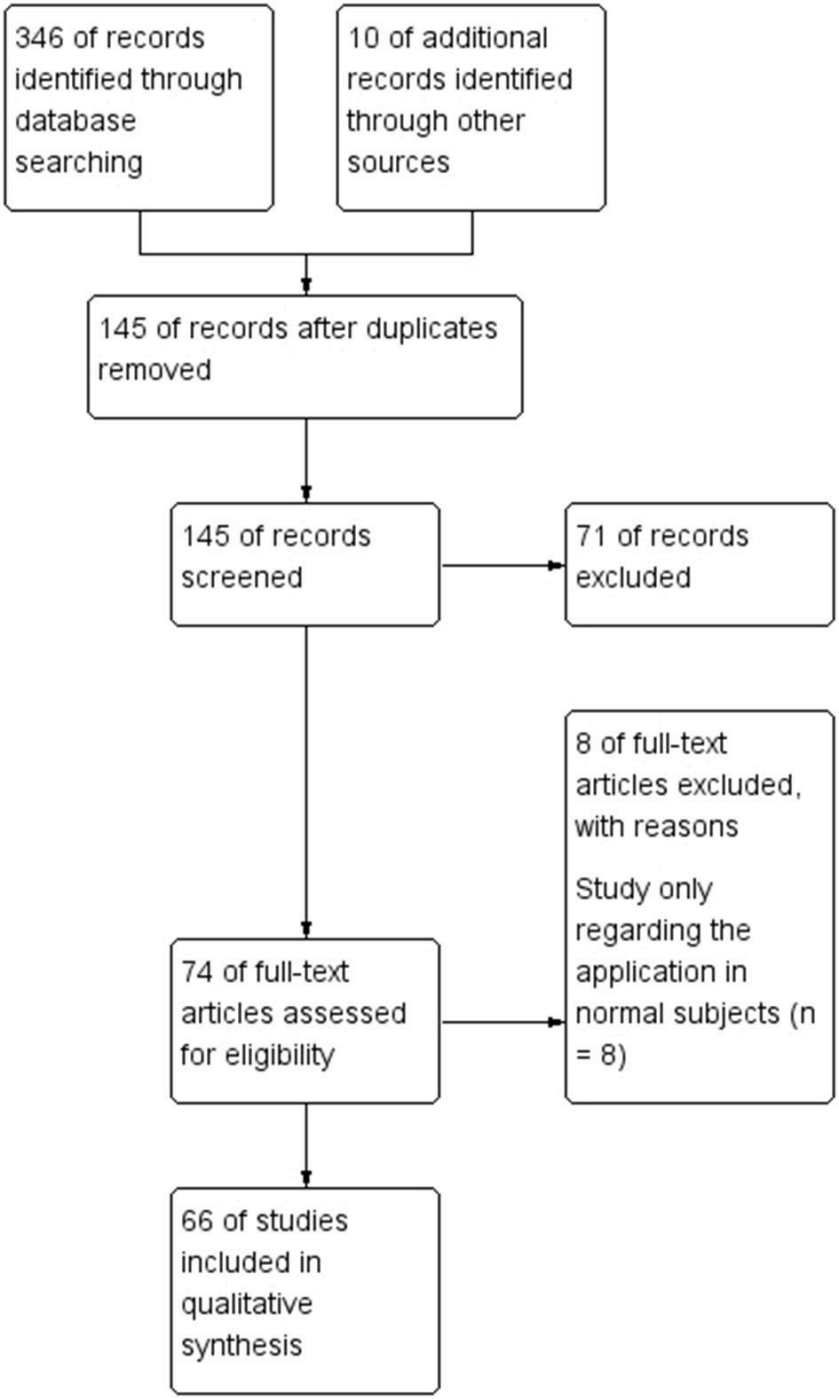
Flowchart of literature search.

**Figure 2:**
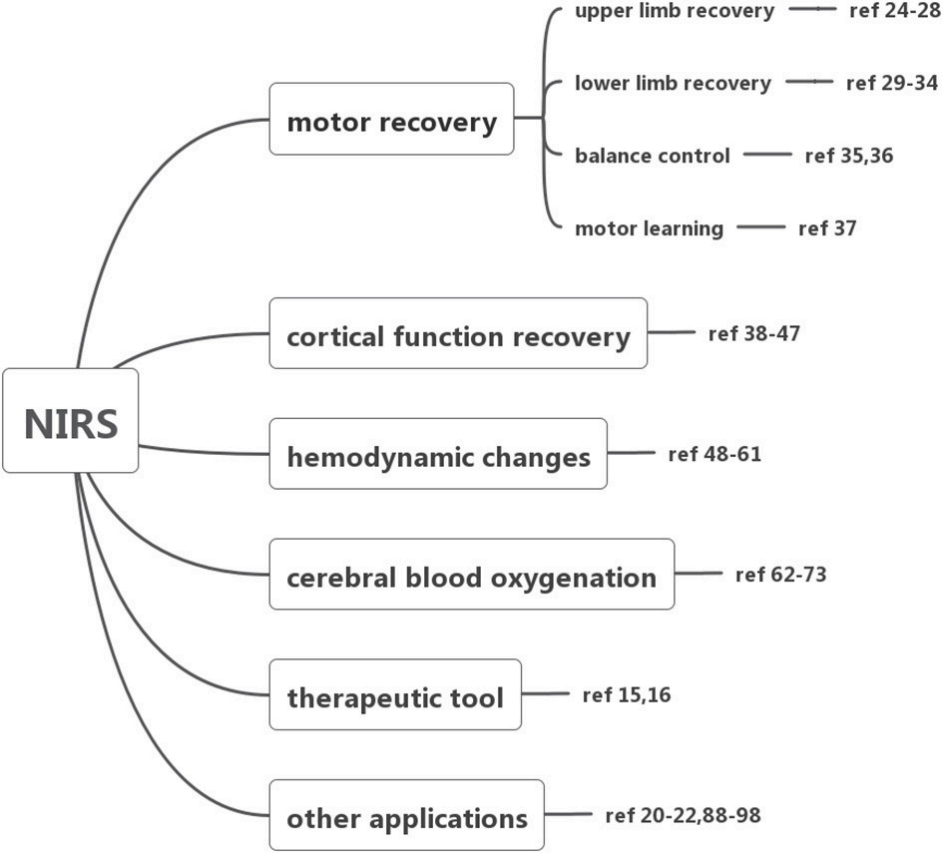
Summary of the application of NIRS.

To evaluate how NIRS has been employed for surveillance and treatment of stroke patients, we conducted a systematic literature review of all published original research involving NIRS in stroke patients. The literature search, conducted in July 2018, began with an initial search of the NCBI Pubmed database using the following search terms: “(‘Spectroscopy, Near-Infrared' [MeSH Major Topic] OR “near-infrared spectroscopy”[Title/Abstract] OR fNIRS[Title/Abstract]) AND (stroke[MeSH Major Topic] OR “cerebrovascular disorders”[MeSH Major Topic] OR “brain infarction”[MeSH Major Topic])”. Additional spot check searches stemming from key references in identified works were also carried out to further strengthen the literature review's reliability. Only English language articles published in peer-reviewed journals were included. Then we searched EMBASE database, Cochrane Library and ScienceDirect database (Figure 1). All studies involving NIRS in stroke patients of any age were included. The details of included studies are presented in Tables 1–7. In total, our literature search identified 66 unique papers relating to stroke recovery, including motor recovery, motor learning, cortical function, hemodynamic changes, cerebral blood oxygen, Near-Infrared Spectroscopy-Based Brain–Computer Interface, and other applications in stroke patients, which were included for further analysis (Figure 2).

**Table 1:** Summary of studies of NIRS in motor recovery.

**Study**	**# of patients**	**Patients character**	**Etiology**	**Target area**	**Measurement**	**Method**	**Clinical applications**
**UPPER LIMB RECOVERY**							
Kato et al. (24)	6	4 men,2 women; 59–79 years old	Cerebral infarction of the middle cerebral artery territory	MCA	Δ[Oxy-Hb] and Δ[Deoxy-Hb].	Multi-channel NIRS	Evaluate the compensatory motor activation of cortical region
Takeda et al. (25)	5	one man, four women, 52–67 years old	Ischemic stroke	Bilateral SM1	Δ[Oxy-Hb] and Δ[Deoxy-Hb].	44-channel NIRS	Monitor cerebral activation and investigate the longitudinal change
Hara et al. et al. (26)	16	14 men, 2 women	Chronic stroke patients with residual hemiparesis	SMC	Δ[Oxy-Hb] and Δ[Deoxy-Hb].	24-channel NIRS	Investigate relationship between hemiparetic arm function improvement and brain cortical perfusion change during different tasks
Gobbo et al. (27)	23	13 males; 10 females, aged 60.4 ± 13.2) years (range: 40–84 years	Subacute or chronic stroke	Flexor muscles of the forearm	Δ[Oxy-Hb] and Δ[Deoxy-Hb].	fNIRS	Evaluate the acute effects induced by a single session of robot-assisted passive hand mobilization on local perfusion and upper limb function
Brunetti et al. (28)	11	Six female, seven male	Subacute stroke with severe upper limb paresis	Primary motor cortex and precuneus	Δ[Oxy-Hb] and Δ[Deoxy-Hb].	fNIRS	Investigate determinants of efficacy of mirror therapy
**LOWER LIMB RECOVERY**							
Miyai et al. (29)	6	Four men, two women; 57 years old	Stroke with hemiplegia	Bilateral frontoparietal cortices covering SMCs, PMCs, SMAs, pre-SMAs, part of prefrontal cortices, and the superior parietal lobes	Δ[Oxy-Hb] and Δ[Deoxy-Hb].	Multi-channel NIRS	Investigate motor cortices activation
Miyai et al. (30)	8	5 men, 3 women; 4 with right and 4 with left hemiparesis; aged 57 years;	Stroke	Bilateral frontoparietal cortices covering the primary SMC, PMC, SMA, pre-SMA, part of PFC, and the superior parietal lobes	Oxy-Hb, deoxy-Hb, and total-Hb levels	Multi-channel NIRS	Illuminate the mechanism of gait recovery
Mihara et al. (31)	12	11 men,1 woman;age range: 12–74 years, mean ± S.D.: 52.7 ± 16.9 years	Infratentorial stroke	Frontoparietal region covering bilateral PFC,the medial PFC,SMA and medial SMC	Δ[Oxy-Hb] and Δ[Deoxy-Hb].	42-channel NIRS	Investigate the difference of cortical activation between patients and controls
Lin et al. (32)	17	16 men, 1 woman; age 55.53 ± 12.06	Subcortical stroke with hemiparesis	Frontoparietal region covering SMC;bilateral SMA and PMC	Δ[Oxy-Hb] and Δ[Deoxy-Hb].	Multichannel frequency domain NIRS	Measure the cortical activation patterns during active cycling with and without speed feedback
Rea et al. (33)	7	6 men, 1 woman	Chronic stroke	Frontal regions, bilateral PMC, SMA, primary and secondary motor areas, and somatosensory areas	Δ[Oxy-Hb] and Δ[Deoxy-Hb].	fNIRS	Assess the reliability and feasibility of NIRS in clinical usability in lower limb movement preparation
Holtzer et al. (34)	236	age ≥ 65 years	167 normal,40 peripheral NGA,29 central NGA	Forehead	Δ[Oxy-Hb] and Δ[Deoxy-Hb].	fNIRS	Confirm the posture first hypothesis
**BALANCE CONTROL**							
Mihara et al. (35)	20	15 men and five women; mean (SD) age 61.6 (11.9) years	Stroke	Frontoparietal region	Δ[Oxy-Hb] and Δ[Deoxy-Hb].	fNIRS	Evaluate cortical activation associated with external postural perturbation
Fujimoto et al. (36)	20	17 men and 3 women; mean (± SD) age 60.2 (± 9.5) years	Subcortical stroke	Frontoparietal region	Δ[Oxy-Hb] and Δ[Deoxy-Hb].	Single-channel NIRS	Evaluate cortical activation associated with external postural perturbation
**MOTOR LEARNING**							
Hatakenaka et al. (37)	12	10 men and 2 women; mean (± SD) age 56 (± 16) years	Ataxia resulting from infratentorial stroke	Forehead, including SMC, SMA, pre-SMA, dorsal PMC, and dorsolateral PFC	Δ[Oxy-Hb] and Δ[Deoxy-Hb].	42-channel NIRS	Investigate impact of the capacity for motor learning on motor recovery

*MCA, middle cerebral artery; SMC, sensorimotor cortex; PMC, premotor cortex; SMA, supplementary motor area; PFC, prefrontal cortex*.

**Table 2:** Summary of studies of NIRS in cortical function recovery.

**Study**	**# of patients**	**Patients character**	**Etiology**	**Target area**	**Measurement**	**Method**	**Clinical applications**
Park et al. (38)	1	Male, 73	Subcortical stroke	Bilateral motor area	Δ[Oxy-Hb] and Δ[Deoxy-Hb].	fNIRS	Assess serial changes during CIT
Schytz et al. (39)	108 (four studies)		Ischemic stroke	MCA	Δ[Oxy-Hb] and Δ[Deoxy-Hb].	Multi-channel NIRS	Assess CA by recording LFO
Han et al. (40)	10	Age: 76.6 ± 8.5 year	Cerebral infarction	Left and right prefrontal lobes	HbO2 signals	fNIRS	Assess the prefrontal functional connectivity using wavelet coherence analysis of cerebral tissue oxyhaemoglobin concentration (Delta [HbO2]) signals in elderly subjects with cerebral infarction (CI) during the resting state
Cao et al. (41)	6	3 male, 3 female; age 10.2 ± 2.1	Stroke with hemiplegia	Sensorimotor cortex, covering PMC, SMA and M1/S1	Oxy-Hb, deoxy-Hb, and total-Hb levels	fNIRS	Evaluate cortical plasticity after CIT
Tan et al. (42)	12	6 men, 6 woman	Cerebral infarction	Bilateral forehead	Δ[Oxy-Hb] and Δ[Deoxy-Hb].	Multi-channel NIRS	Evaluate functional connectivity
Oldag et al. (43)	20	12 men, 8 women; mean age 55.3 ± 12.5 years	Unilateral high-grade steno-occlusion of MCA	Bilateral MCA	Δ[Oxy-Hb] and Δ[Deoxy-Hb].	Multi-channel continuous wave NIRS	Assess autoregulation delay
Moriya et al. (44)	11	Seven males, four females, aged 69.6 ± 12.0 years	Stroke	Prefrontal cortex	Δ[Oxy-Hb] and Δ[Deoxy-Hb].	Two-channel NIRS	Examine the acute effect of physical exercise
Saita et al. (45)	2	58-year-old male; 74-year-old female	Cerebellar stroke	Bilateral frontal lobe	Δ[Oxy-Hb] and Δ[Deoxy-Hb].	fNIRS	Evaluate cortical activity
Mori et al. (46)	14	12 men and 2 women; mean age, 61.1 ± 9.3 years; range, 36–72 years	Stroke	Prefrontal cortex	Δ[Oxy-Hb] and Δ[Deoxy-Hb].	16-channel NIRS	Investigate the association between PFC activity and dual-task interference on physical and cognitive performance
White et al. (47)	2	Infant	Perinatal stroke	Bilateraloccipital Lobe	Oxygenation	NIRS oximetry	Investigate the functional organization of the brain in adults and infants

*CIT, constraint-induced therapy; CA, cerebral autoregulation; LFO, low-frequency oscillations*.

**Table 3:** Summary of studies of NIRS in monitoring hemodynamic changes.

**Study**	**# of patients**	**Patients character**	**Etiology**	**Target area**	**Measurement**	**Method**	**Clinical applications**
Saitou et al. (48)	44		Stroke with hemiplegia	Forehead on the injured side			Detect cerebral perfusion
Terborg et al. (49)	13	Five female, eight male; mean (SD)age, 62.2 (13.0) years	Acute infarction in the territory of MCA	The territory of the MCA	Kinetics of an intravenous bolus of indocyanine green	NIRS	Detect cerebral ICG kinetics
Terborg et al. (50)	11	6 male, 5 female; mean age, 65.8 ± 12.4 years	Acute infarction in the territory of MCA	The territory of the MCA	Kinetics of an intravenous bolus of indocyanine green	NIRS	Measure effects of head-of-bed (HOB)
Durduran et al. (51)	17	7 male, 10 female; 44-93 years (65 ± 16)	Acute ischemic stroke	Frontal lobe	Δ[Oxy-Hb] and Δ[Deoxy-Hb].	Two-channel NIRS	Monitor blood flow signals
Muehlschlegel et al. (52)	12	8 male, 4 female; 49-89 years	Hemispheric strokes	Injured area	Interhemispheric correlation coefficient (IHCC)	CW-NIRS	Assess the cerebral oxygenation oscillations
Li et al. (53)	10	7 male,3 female; age 65 ± 7	CI	Frontal lobe	Cerebral oxygenation oscillations	NIRS oximetry	Monitor neuronal and vascular signals
Leistner et al. (54)	6	3 male,3 female; age 52 ± 5	Subacute ischemic stroke	Frontal lobe	Δ[Oxy-Hb] and Δ[Deoxy-Hb].	fNIRS	Monitor blood flow signals
Keller et al. (55)	1	_	Subarachnoid hemorrhage	Injured area	Kinetics of an intravenous bolus of indocyanine green	NIRS	Detect cerebral perfusion
Oldag et al. (56)	17	59.9 ± 13.8	Severe unilateral stenosis or segmental occlusion of the MCA	The cortical territory of the MCA and its border zone toward the anterior cerebral artery	Δ[Oxy-Hb] and Δ[Deoxy-Hb].	Multichannel continuous wave NIRS	Assess the phase relationship of prefrontal tissue oxyhemoglobin oscillations
Han et al. (57)	21	74.4 ± 9.0 years	CI	Frontal lobe	Δ[Oxy-Hb] and Δ[Deoxy-Hb].	fNIRS	Assess changes at microvascular level
Zirak et al. (58)	1		Ischemic stroke	/			Assess neurovascular coupling
Dutta et al. (59)	4	3 males, 1 female from age 31 to 76	Chronic ischemic stroke	Central site Cz	Oxy- and deoxy- hemoglobin	NIRS oximetry	Investigate hemodynamic and metabolic changes
Mitra et al. (60)	1	Infant	Perinatal stroke	Either side of forehead	Oxy- and deoxy- hemoglobin	NIRS oximetry	
Cooper et al. (61)	4	Infant	Perinatal stroke	Each temporal lobe	Oxy- and deoxy-hemoglobin	NIRS oximetry	Investigate transient hemodynamic events within few hours in infants with neurological damage

*CI, cerebral infarction; ICG, indocyanine green*.

**Table 4:** Summary of studies of NIRS in monitoring cerebral blood oxygenation.

**Study**	**# of patients**	**Patients character**	**Etiology**	**Target area**	**Measurement**	**Method**	**Clinical applications**
Nemoto et al. (62)	2	85-yr-old female; 62-yr-old female	Infarction in the territory of MCA	Frontal-temporal or frontal-parietal cortices	Oxygen saturation	NIRS oximetry	Monitor CBO changes
Damian et al. (63)	24	15 male, 9 female; 43-77	Complete MCA stroke and brain swelling	Bilateral frontal lobe	Oxygen saturation	NIRS oximetry	Monitor CBO changes
Sakatani et al. (64)	/	/	/	/	Oxygen saturation	NIRS oximetry	Clarify CBO changes occurring in stroke and brain tumors
Aries et al. (65)	9	3 male, 6 female; age 71 ± 10	Acute stroke	Bilateral forehead	Oxygen saturation	NIRS oximetry	Monitor CBO changes
Mundiyanapurath et al. (66)	44	59–82	Acute ischemic stroke		Oxygen saturation	NIRS oximetry	Compare therapeutic effect
Liebig et al. (67)	/	/	/	/	Cerebral oxygenation	NIRS oximetry	Compare therapeutic effect
Hametner et al. (68)	43	22 male, 21 female; 65–79	Acute ischemic stroke	Forehead	Cerebral oxygenation	NIRS oximetry	Measure rSO2
Hashimoto et al. (69)	1	77-year-old man	CI	Bilateral frontal lobe	Regional cerebral oxygen saturation (rSO2)	NIRS oximetry	Monitor CBO changes
Ritzenthaler et al. (70)	3	56-year-old man; 52-year-old man; 40-year-old pregnant woman	MCA occlusion	Bilateral forehead	Regional cerebral oxygen saturation (rSO3)	NIRS oximetry	Assess rSO2 changes
Nakamura et al. (71)	5	Four male, one female, 65.6 ± 10.6 years	Stroke	Injured area	Δ[Oxy-Hb] and Δ[Deoxy-Hb].	NIRS	Evaluate effect of revascularization on the abnormal evoked CBO responses
Vernieri et al. (72)	2	58-year-old man; 61-year-old man	Ischemic stroke	MCA	Cerebral oxygenation	NIRS oximetry	Evaluate the effects of hypercapnia
Moreau et al. (73)	5	2 male,3 female; median age 64 years old	Acute stroke	MCA	Cerebral oxygenation	NIRS oximetry	Investigate brain oxygenation in the first few hours of stroke onset

*CBO, cerebral blood oxygenation*.

**Table 5:** Summary of studies of NIRS as therapeutic tool.

**Study**	**# of patients**	**Etiology**	**Target area**	**Clinical applications**
Blokland et al. (15)	10	Stroke with tetraplegia	Motor cortex	Test the feasibility of a combined EEG-fNIRS system
Mihara et al. (16)	20	Subcortical stroke	Frontoparietal region	Investigate the effect of neurofeedback on motor imagery

**Table 6:** Summary of studies of NIRS in evaluating the risk for perioperative stroke.

**Study**	**# of patients**	**Operation**
Olsson et al. (74)	46	Selective antegrade cerebral perfusion
Nakamura et al. (75)	1	CEA
Kellermann et al. (76)	/	Cardiac surgery
Pennekamp et al. (77)	/	Carotid endarterectomy
Ono et al. (78)	234	Cardiopulmonary bypass
Rummel et al. (79)	35	Balloon occlusion

*CEA, Carotid Endarterectomy*.

**Table 7:** Summary of studies of other applications.

**References**	**# of patients**	**Etiology**	**Clinical applications**
**THE RISK FOR STROKE**			
Viola et al. (80)	10	Migraine	Detect cerebral microcirculation
Li et al. (81)	12	Atherosclerosis	Evaluate risk for atherosclerotic stroke
Pizza et al. (82)	11	Acute/subacute middle cerebral artery stroke	Monitor oxygenation level
Liebig et al. (67)	25	Ischemic stroke	Monitor signs of ischemia during neurovascular procedures
**RESEARCH TOOL**			
Bönöczk et al. (20)	43	Middle cerebral artery stroke	Measure cerebral blood flow
De et al. (21)	3	Perinatal arterial ischemic stroke	Assess cerebral oxygenation
**OTHERS**			
Selb et al. (83)	36	Stroke	Investigate the effect of MAs on “oscillation” data
Ferrante et al. (22)	9	Stroke	Examine muscular metabolism

*MA: motion artifacts*.

## NIRS for Motor Recovery

We identified 14 papers (24–37) reporting on NIRS for motor recovery in a total of 399 patients, including upper limb function recovery, lower limb function recovery, balance control and motor learning (Table 1). In most patients, their motor function was significantly injured due to stroke, resulting in hemiparesis or tetraplegia. The concentration of oxy-Hb, deoxy-Hb, and total Hb were calculated by NIRS during various tasks, reflecting the activity of motor cortex. The probe is usually positioned at frontal regions, covering motor cortex, including bilateral premotor cortex (PMC), supplementary motor area (SMA), primary and secondary motor areas, and somatosensory areas.

Five papers (24–28) reported upper limb motor recovery (Table 1). In three of them, NIRS reflect cerebral cortical activity according to blood flow signals. Upper limb motor recovery has been shown to be associated with ipsilateral motor cortical compensation. Kato et al. evaluated the compensatory motor activation of cortical regions in 6 patients with cerebral infarction and 5 normal controls in comparison with functional MRI (fMRI) and demonstrated the contribution of ipsilateral motor cortical compensation or reorganization during recovery (24). Moreover, patients have shown longitudinal changes in functional laterality of activations in the primary sensorimotor cortex that at early stage affected hand movements activated bilateral sensorimotor cortices, whereas the activation pattern returned toward normal at later periods (25). Local perfusion may also play an essential role in motor recovery. Hara et al. studied the relationship between improvement in arm function and cerebral cortical perfusion changes during different tasks or treatments, in which electromyography-controlled functional electrical stimulation (EMG-FES) significantly increased cerebral blood flow, and may improve upper limb function (26). In another paper, the probe was placed on the flexor muscles of the forearm to detect changes in forearm tissue perfusion a single session of robot-assisted passive hand mobilization in 23 patients with subacute or chronic stroke patients. The results showed decreased upper limb heaviness and stiffness due to improved local circulation, therefore providing evidence that hand robotic assistance may help in the management of spasticity, heaviness, stiffness, and pain in patients with post-stroke hemiparesis (27). In addition, Mirror therapy (MT) has been shown that it could significantly improve upper limb motor function despite individual variance (28).

We found six papers (29–34) describing gait control and lower limb function recovery (Table 1). Miyai et al. demonstrated the contribution of motor cortices including PMC and pre-SMA in restoration of gait in patients with severe stroke (29). They then illuminated more detailed mechanism of gait recovery (30). By measuring cortical activities during hemiparetic gait on the treadmill, this study discovered gait improvement of asymmetry in medial primary sensorimotor cortex (SMC) activation and enhanced PMC activation in the affected hemisphere may play a role in locomotor recovery. However, the activation between stroke patients and normal subjects may be diverse. Another study further investigated the difference of cortical activation between patients and controls. The results revealed that during the acceleration phase both controls and patients showed obvious cortical activation in the medial SMC, SMA and prefrontal cortex (PFC). However, in the steady phase, brain activation attenuated in controls, but persisted in patients (31). Lin et al. compared cortical activation patterns during active cycling with and without speed feedback and during power-assisted cycling in stroke patients. Speed feedback may enhance the PMC activation and improve cycling performance in stroke patients (32). The reliability and feasibility of NIRS in monitoring lower limb movement recovery has been proved (33). Holtzer et al. conducted a research to confirm the posture first hypothesis that under dual-task walking conditions older adults prioritize gait over cognitive task performance. They assessed oxy-Hb levels in the PFC during normal walk and walk while talk (WWT) conditions in a large cohort of non-demented older adults, including normal subjects, central and peripheral neurological gait abnormalities (34). Interestingly, higher oxygenation levels were associated with better cognitive performance among normals, whereas, faster gait velocity in peripheral neurological gait abnormalities (NGA). This result suggested that presence of NGA may influence distribution of brain activities between gait and cognitive performance (34).

Balance problem is one of the major sequelae of stroke. Two papers (35, 36) focused on the mechanism of postural balance with external perturbation (Table 1). Mihara et al. Revealed broad cortical area, including the prefrontal, premotor, supplementary motor, and parietal cortical areas in both hemispheres, involved in balance control in poststroke patients (35). Among which, SMA was then demonstrated to be a crucial area—the increment of the postural-perturbation-related oxygenation signals in the SMA of the unaffected hemisphere was significantly correlated with the gain in balance function (36).

Motor learning is essential in function recovery, including motor adaptation, and motor sequence learning. Motor adaptation refers to the ability to compensate for environmental changes such as external force. Motor sequence learning is defined as learning of sequential motor actions, which is essential for activities of daily living. Impaired motor sequence learning may be correlated with reduced rehabilitation gains for ataxic patients with stroke (37).

## NIRS for Cortical Function Recovery

The cortical functions including cortical plasticity, functional connectivity or cerebral autoregulation that could be reflected based on oxygenation levels are directly related to motor and cognitive recovery after stroke. We reviewed 10 papers (38–47) reporting cortical function recovery (Table 2).

Two studies have reported significant improvement in task performance or cortical plasticity after constraint-induced movement therapy (38, 41). In addition, fNIRS could assess the prefrontal functional connectivity using wavelet coherence analysis of cerebral tissue oxy-Hb concentration (40). A frequency-specific disruption in resting-state connectivity has been observed in cerebral infarction cases. fNIRS could also used in neonates as a bedside monitoring tool to early detect neurological deficiency and provide prognostic information (47). These evidence supported NIRS as a promising method for evaluating functional recovery (42).

Post-stroke patients exhibit changes in cerebral autoregulation (CA) which could be assessed by recording spontaneous oscillations in mean arterial blood pressure and cerebral blood flow. The etiology of oscillations remained unknown, however, its significance in reflecting CA has been demonstrated. NIRS with a high time resolution is suggested as a promising tool for measuring oscillations, identifying risk and evaluating treatment strategies in patients with carotid artery disease and ischemic stroke (39, 43).

Mori et al. investigated dual-task interference on physical and cognitive performance in stroke patients. In consistent with previous studies, during dual-task walking, PFC activation might prioritize physical demands in stroke patients, but might prioritize cognitive demands in healthy subjects (46). Another study examined the acute effect of physical exercise on prefrontal cortex activity in post-stroke patients and demonstrated the therapeutic effect of moderate-intensity aerobic exercise by improving working memory performance (44).

## NIRS for Hemodynamic Changes

Fourteen papers (48–61) focusing on the applications of NIRS in monitoring hemodynamic changes in 158 stroke patients (Table 3). The probe usually positioned on the injured area of the brain, NIRS can detect cerebral perfusion timely in patients with acute infarction (49, 50) or steno-occlusive disease (56) at bedside with indocyanine green (ICG) as tracer, providing a rapid, repeatable method without major side effects and transportation of critically ill patients (48–50, 56). Another study explored Diffuse correlation spectroscopy (DCS) and NIRS to measure effects of head-of-bed (HOB) positioning at different angles. The result showed increase in cerebral blood flow (CBF) when the HOB is lowered from 30° to −5° (51). Mitra et al. investigated metabolic and circulation changes following neonatal stroke in a term infant. Decreased oxy-Hb and clear asymmetry were noted (60).

NIRS could be used in intensive care unit as a neuromonitoring tool, reflecting blood flow signals (52, 55) as well as neuronal and vascular signals (54) in real-time. The interhemispheric correlation coefficient (IHCC) during physiological oscillations might be a new NIRS analysis tool to quantify asymmetric microvascular hemodynamics (52). In addition, NIRS combined with diffuse correlation spectroscopy might provide hemodynamic changes at the microvascular level (58).

Spontaneous activity in prefrontal cerebral oxygenations has been demonstrated to be disturbed in the elderly and in patients with cerebral infarction (CI), which could be analyzed by continuous recording based on the wavelet transform of NIRS signals, could reflect resting state functional connectivity (53). Recent studies have shown the reduction of spontaneous oscillations in subjects with CI may suggest an increased stiffness in arterial vessels (53). Moreover, altered phase synchronization has been observed between the CI patients and healthy elderly indicates altered phase synchronization, indicating its potential utility to assessing atherosclerosis in high risk subjects for CI (57).

Hemodynamic changes recorded by NIRS could also provide critical information for other techniques. A method for EEG-NIRS based assessment of neurovascular coupling (NVC) during anodal transcranial direct current stimulation (tDCS) was proposed in case series of four chronic ischemic stroke survivors (59). Cooper et al. used this EEG-NIRS system recorded transient haemodynamic events in neurologically compromised infants (61).

## NIRS for Cerebral Blood Oxygenation

We reviewed twelve (62–73) papers reporting on cerebral blood oxygenation (CBO) (Table 4). NIRS allows non-invasive measurement of regional cerebral oxygen saturation with high time resolution, reflecting cerebral perfusion and metabolism.

Oximetry by NIRS evaluates oxygenation changes in stroke patients (73) and the balance between regional oxygen supply and demand and assess functional state of the brain. In infarcted non-metabolizing brain, oxygen saturation may be near normal due to sequestered cerebral venous blood in capillaries and decreased consumption. In regionally or globally ischemic, but metabolizing brain, saturation decreases because oxygen supply is insufficient to meet metabolic demand (62). Several studies demonstrated good responsiveness of NIRS signal to oxygenation changes in stroke and brain tumors (63–65). Unlike fMRI which provides information mainly about concentration changes of deoxy-Hb that is paramagnetic,. NIRS detect both oxy-Hb and deoxy-Hb changes, thus providing more information (64). In addition, bilateral NIRS seems to be more useful on cerebral oxygenation than unilateral measurements and may help in prediction of the worsening of brain swelling (63).

One of the most important predictors of clinical outcome after acute ischemic stroke is recanalization. Endovascular treatment is one of major therapies of stroke patients. NIRS may be useful to monitor oxygenation during management of acute ischemic stroke (70). Nakamura et al. demonstrated that revascularization could improve the abnormal evoked-CBO response in ischemic stroke patients (71). Studies were designed to optimize clinical treatment with NIRS as a monitoring tool in measuring cerebral oximetry. However, whether patients suffering from acute ischemic stroke and undergoing endovascular recanalization should be treated under general anesthesia (GA) or conscious sedation (CS) remained a matter of debate. A prospective study with forty-four patients investigated their effects. The result showed CS groups required less vasopressor medication and had a higher mean blood pressure (66). Mechanical thrombectomy might have higher recanalization rates than intravenous or intra-arterial thrombolysis, resulting in a better clinical outcome (67). Treatment of endovascular stroke often impedes neurologic assessment in patients who need sedation or general anesthesia. NIRS may fulfill this gap by measuring regional oxygen saturation (rSO2). Bi-channel rSO2-NIRS has potential for guiding neuroanesthesia and predicting outcome (68).

Cerebral hyperperfusion syndrome (CHS) was described in a case report of a 77-year-old man with acute cerebral infarction, which is known to be a rare but devastating complication of carotid artery revascularization. The patient underwent successful recanalization with thrombectomy by using a stent retriever for the middle cerebral artery and stent placement for the origin of the internal carotid artery. However, 12 h after treatment acute intracranial hemorrhage happened, which was considered to have been caused by CHS under the evaluation of NIRS. This case emphasized the significance of performing routine monitoring of regional cerebral oxygen saturation by NIRS to timely recognize CHS (69).

NIRS may be more useful in combination with other techniques. Vernieri et al. combined NIRS with transcranial Doppler to investigate the effects of hypercapnia on healthy individuals and patients of ischemic stroke (76). The results revealed that in healthy hemisphere scalp vessels dilated in response to hypercapnia, thus increasing cerebral blood flow, whereas in ischemic side this changes was negligible. This hybrid method could provide more information, with transcranial Doppler recording subcortical changes and NIRS—cortical arterioles and capillary modifications (72).

## NIRS as a Therapeutic Tool

Despite several efficient therapies, neurorehabilitation is an intricate process requiring time and numerous factors. Given this circumstance, BCI which use brain activity to control external devices may be the only solution for patients with severe function deficiency, failing to interact with the environment and sustain normal daily lives.

BCI can be divided into two types, assistive BCI and rehabilitative BCI system. The assistive BCI system aims to substitute lost functions, for example, to achieve high-dimensional movement control of robotic devices. In contrast, the rehabilitative BCI system aims to facilitate the restoration of brain function by normalizing the neurophysiologic activity (84). The patient's brain signals obtained via invasive or non-invasive means are acquired via amplifiers, filtered, and decoded using an online classification algorithm to control external devices. Distinct techniques have been explored to control a BCI, such as EEG and more recently NIRS.

NIRS is a relatively new measurement modality that offers a portable, low-cost, sensitive option for BCI development. The idea of using NIRS as an optical BCI has been introduced by Coyle in 2004 (85). Since then a number of studies (84–90) further investigated its application in BCI, either examining the resulting signals for motor imagery or classifying the NIRS signals directly. Despite significant advantages because of non-invasive, portable nature of NIRS, there are several seconds of delay between neural activation and NIRS signal changes. To overcome these disadvantages of NIRS-based BCI systems, Siamac et al. combined EEG and NIRS measurements in BCI (91). Recently this hybrid EEG–fNIRS brain computer interface has attracted extensive attention and shown enhanced accuracy (92–97). Most of these studies, however, are limited in healthy population. Blokland et al. demonstrated feasibility and accuracy of using a combined EEG-fNIRS sensorimotor rhythms-based brain switch in patients with tetraplegia. A combined EEG-fNIRS system might be especially beneficial for patients whose classification performance based on EEG only are low (15). The recent study of Khan et al. Has shown the possibility of this hybrid EEG-fNIRS system in controlling a quadcopter with fNIRS decoding mental arithmetic, counting, rotation, and word formation, EEG decoding eyeblinks and movement (92). The combination of EEG and fNIRS increases the number of control commands, improve classification accuracy and reduce the signal detection time (93). Further improvement in its efficacy requires proper brain region identification and feature selection (94).

In addition, NIRS was used in neurofeedback. Neurofeedback is a type of biofeedback that measures brain waves to produce a signal that can be used as feedback to modulate brain function voluntarily by the patients themselves. Real-time feedback of neural activity to the subjects may modulate plastic reorganization without external stimulation of the brain. NIRS, as a neurofeedback tool detecting blood flow signals, could improve cortical plasticity and enhance the therapeutic effect of motor imagery. In a pilot study twenty hemiplegic patients with subcortical stroke were analyzed and the results suggested that NIRS-mediated neurofeedback may enhance the efficacy of mental practice with motor imagery and augment motor recovery (16).

## Other Applications

Apart from applications above, NIRS has been used in evaluation of the risk for stroke, clinical studies and monitoring muscle metabolic indexes (Tables 6, 7).

Continuously monitoring oxygenation levels by NIRS during operation could diagnose and predict the occurrence of perioperative stroke. In a study of 46 patients underwent selective antegrade cerebral perfusion of the right subclavian artery, decreased regional cerebral tissue oxygen saturation was observed. Monitoring of regional cerebral oxygenation by NIRS allows detection of clinically important cerebral desaturation, and could predict perioperative neurologic sequelae (74). Two studies evaluated perioperative stroke after carotid endarterectomy (75, 77). NIRS could monitor cerebral changes during the surgery, providing immediate feedback to the treating physician and allowing prompt corrections. Multichannel NIRS detects watershed-type posterior perfusion defects, superior to single-channel (75). NIRS is also of great importance during cardiopulmonary bypass (78), cardiac surgery (76), and vessel occlusion (67, 79). Treatment of intracranial aneurysms by surgical clipping carries a risk of intraoperative ischemia, caused mainly by prolonged temporary occlusion of cerebral arteries. NIRS monitoring of cerebral blood flow (CBF) during surgery may decrease the risk (67). Another study proposed multichannel CW NIRS during endovascular neuroradiologic interventions requiring temporary balloon occlusion of arteries supplying the cerebral circulation (79). Based on these studies, NIRS plays an essential role in monitoring perioperative stroke, however, a combination of several monitoring modalities may enhance accuracy and require further investigations.

Studies have also evaluated the risk for stroke in other diseases. We reviewed three papers. Viola et al. assessed hemodynamic changes in migraine patients. The results showed a mild vasoconstriction, which may be the possible explanation of the association between migraine and an increased risk for ischemic stroke (80). The spontaneous cerebral oscillations based on the wavelet transform of NIRS signals were assessed to evaluate risk for atherosclerotic stroke (81). Sleep-disordered breathing (SDB) could lead to cerebral deoxygenation, resulting in stroke outcome. By monitoring oxygenated state of the brain, NIRS could recognize these conditions at early stage (82).

In addition, NIRS presents as an effective research tool in clinical studies. The concentration of oxy-Hb and deoxy-Hb obtained by NIRS could be measured, calculated and analyzed, evaluating hemodynamic, oxygenation changes and cortical function, thus widely used in assessing the effects of different therapies or the evolution of diseases (20, 21). Ferrante et al. combined functional electrical stimulation and time-domain NIRS (TD NIRS) to examine muscular metabolism on post-stroke patients and the result indicated metabolic dysfunction of muscles seemed to be local and unilateral (22).

## Discussion and Future Directions

NIRS, a non-invasive neuroimaging technique with the advantages of low price, simplicity, portability and small devices, has a wide range of utility (48). The existing clinical literature suggests NIRS plays an important role in monitoring motor recovery, including upper limb (24–28), lower limb recovery (29–34), balance control (35, 36), motor learning (37), cortical function recovery (38–47), cerebral hemodynamic changes (48–61), cerebral oxygenation (62–73), therapy (15, 16), and other applications (20–22, 67, 74–83). In particular, its ambulatory real-time measurement without the fixation of position provides great value in tasks requiring constant movement, for example, gait control.

NIRS is applied in healthy population at first and then used in stroke subjects. By monitoring of NIRS could reflect the function recovery and the therapeutic effect in patients; compared with normal people, it can be used to study the pathophysiological mechanism of disease and predict the risk, serving as an effective monitoring and research tool. In addition, it can be used as therapeutic tool combined with BCI and neurofeedback, but the current studies mainly limited in healthy people, however, the results have shown potential value, requiring further investigations in stroke patients.

Despite its non-invasive, portable and ambulatory nature, its accuracy is influenced by several factors, thus limiting its further application. fNIRS detects neurovascular coupling and its accuracy may be impaired by extracerebral and systemic changes such as blood pressure and concentration of CO2 (98, 99). This problem confounds all hemodynamic-based neuroimaging methods, like fMRI. However, due to its high sensitivity, the slight blood fluctuations in the scalp may cause “false positives and negatives.” We generally assumed the signal changes are only related to neural activity. However, in reality, the fNIRS signals are composed of six components: evoked/non-evoked cerebral neuronal changes and evoked/non-evoked cerebral/extracerebral systemic changes (23, 100). These shortages could be minimized by careful study design, improved fNIRS techniques and statistical processing (23). A modified fNIRS techniques with higher resolution is needed. The newly proposed fNIRS-based bundled-optode method used 32 optodes could increase spatial resolution and this method can be extended for 3D fNIRS imaging (101, 102). As to obtain higher temporal resolution for fNIRS-BCI, several studies demonstrated the detection of the initial dip may be useful. The initial dip refers to an early small decrease of the concentration of oxy-Hb and is closely associated with neuronal metabolism. fNIRS-based BCI using initial dip detection could reduce the command generation time and improve temporal resolution (103, 104). In addition, longitudinal studies, for example, measuring with NIRS/fNIRS on one subject every day for a week could provide deeper insight the dynamic changes of brain, and as time prolonged the influence of confounding factors could be minimized. Furthermore, stroke has specific circadian and circannual rhythms. Most of strokes occurred in the morning between 7 a.m. and noon (105). Therefore, the time of measurement may influence the efficacy of NIRS and determining the optimal time window for measurements might be one aspect for optimization of NIRS. Apart from “false positives and negatives,” NIRS provides no anatomical information, thus must use scalp anatomy to locate the position where the signals arise (52, 106). At present, inhibitory and excitatory activity cannot be discriminated, which requiring the support of other techniques (107).

Considering the inevitable shortcomings, one promising prospect is the combination of different neuroimaging techniques, including diffusion-tensor imaging (DTI), magnetic resonance spectroscopy (MRS), ligand-based positron emission tomography (PET), single-photon emission computed tomography (SPECT), functional magnetic resonance imaging (fMRI), NIRS, and EEG. Conjugated with other techniques, the surveillance might be more comprehensive and accurate. Moreover, at present most studies have focused on its application in stroke recovery, its ability to assess the occurrence of stroke, for example, during operation and in people with higher risk, is of great significance. Further researches could emphasize NIRS in the prevention of stroke as well as stroke recovery and prognosis.

NIRS could also used in combination with neurostimulation tools. Medical communities have been interested in electrical stimulation for a long time. Recently transcranial direct current stimulation (tDCS), which was firstly used in humans in 2,000 by Nitsche et al. (108), has shown to be an appealing therapeutic tool in neurological disorders. It emits small amounts of electric current to modulate the excitability of neuron and has shown to be effective in many neurological disorders, including stroke, Parkinson's disease, schizophrenia, depression and addiction (109). Several studies has suggested its ability to change oscillatory neural activity in resting state, functional connectivity and event related desynchronization/synchronization which are often injured in patients with motor disorders (110). It could also influence membrane potentials of pyramidal neurons and induce long lasting effects on cortical plasticity by modifying N-methyl-D-aspartate (NMDA) receptor and revealed changes in resting state oscillatory neural activity, functional connectivity, and event related activity during cognitive tasks (111, 112). These results could partially explain its therapeutic effects, but its exact mechanism still remain unclear. Neuroimaging tool, including fMRI, PET, EEG and NIRS could partially illuminate the neurophysiological changes, providing valuable insights into brain changes during electrical stimulation. However, the application of fMRI and PET are restricted due to bulky and immobile properties. EEG are portable and suitable in combination with tDCS, but its accuracy may be diminished by disturbance of artifacts. fNIRS, based on the measurements of light intensity, are not affected by electrically induced artifacts and might be most desirable tool in tDCS researches (113).

## Author Contributions

PW contributed to the conception of the study. MY contributed significantly to manuscript preparation and wrote the manuscript. ZY, TY, and WF revised the manuscript and approved the final version.

### Conflict of Interest Statement

The authors declare that the research was conducted in the absence of any commercial or financial relationships that could be construed as a potential conflict of interest.
